# Predisposing Factors for Erosive Tooth Wear in Permanent Teeth Among Asthmatic Children and Adolescents

**DOI:** 10.1002/cre2.70142

**Published:** 2025-05-07

**Authors:** Tomi Ujčič Samec, Janja Jan

**Affiliations:** ^1^ Department of Dental Diseases and Endodontology, Faculty of Medicine University of Ljubljana Ljubljana Slovenia; ^2^ Centre for Dental Diseases, Division of Stomatology University Medical Centre Ljubljana Ljubljana Slovenia

**Keywords:** asthma, asthma medications, erosive tooth wear, paediatric dentistry

## Abstract

**Objectives:**

Epidemiological studies indicate that erosive tooth wear (ETW) is a common threat of tooth surface loss. The etiology of ETW is multifactorial. The prevalence of asthma is increasing in developed countries, especially in children. Studies evaluating ETW in asthmatic children are conflicting. With our study, we aimed to investigate the association between general and asthmatic factors and the presence of ETW.

**Methods:**

Population of this cross‐sectional observational study consisted of children aged 6–17 years under treatment for asthma at University Medical Centre. ETW was determined using the Basic Erosive Wear Examination index. Questionnaires completed by parents and data from patients' medical records provided information on demographics, medical history, medication use, dietary habits, oral hygiene, fluoride exposure and type, dose, frequency, duration, and mode of asthma medication use.

**Results:**

379 asthmatic children participated in the study. The prevalence of ETW was 17.2% (*n* = 379). The mean total BEWE score was 0.76 ± 2.12. A statistically significant higher presence of ETW and higher total BEWE index were found in the group consuming acidic sports drinks (OR = 3.318), in the group aged 12–17 years (OR = 6.233), in the group using asthma medication for more than 3 years (OR = 3.379) and in the group using medication in the dry powder inhaled form (OR = 2.447).

**Conclusions:**

Asthmatic children should avoid drinking acidic drinks since longer duration of asthma medication use is already associating them with higher ETW presence. From the ETW point of view, metered‐dose inhaled medications are more tooth‐friendly than dry powder inhaled forms.

**Abbreviation**
BEWE: basic erosive wear examiniation

## 1 Introduction

In addition to dental caries, the tooth wear has emerged as an increasingly significant concern within the field of dentistry. Erosive tooth wear (ETW) is defined as irreversible loss of tooth structure due to acid dissolution without the involvement of bacteria and is now considered as the predominant factor contributing to tooth wear (Carvalho et al. 2015). Epidemiological studies alongside in vitro and in situ investigations suggest that ETW is becoming a prevalent threat for tooth surface loss (Korkmaz and Kaptan 2020). There is a wide variation in the global prevalence of ETW, ranging from 0% to 100%. A rough mean calculated from the available data is estimated to be between 20% and 45% for permanent teeth (Schlueter and Luka 2018). The etiology of ETW is multifactorial and includes chemical, biological, and behavioral factors. The acids that attack the tooth structure are intrinsic (gastric acid) or extrinsic (dietary acids, acidic medication, occupational and environmental acids). Among the many risk factors we also find asthma (Donovan et al. 2021).

Asthma is a chronic lung disease characterized by reversible airway obstruction, airway inflammation, and increased airway responsiveness to stimuli. The prevalence of asthma is increasing in industrialized countries, especially in children (Papi et al. 2018). In Slovenia, the prevalence of asthma is estimated to be 14% among children (Kopriva 2003), while in adults it decreases to 2.12% (Poplas‐Susič et al. 2015). The pharmacological management of chronic asthma in pediatric populations typically involves two primary categories of medications: bronchodilators and anti‐inflammatory agents. Children with mild asthma often receive treatment solely with inhaled β2‐agonists as bronchodilators. Inhaled glucocorticoids are recommended for use in children experiencing moderate to severe asthma due to their effectiveness as anti‐inflammatory agents (Papi et al. 2018).

Asthma medications have the side effect of reducing salivary flow (Alaki et al. 2013), they can contribute to the dissolution of the enamel of the teeth they come into contact with and they can influence the development of gastroesophageal reflux disease, all of which can lead to the formation of ETW (Barron et al. 2003; Gani et al. 2020).

While the correlation between asthma and dental caries is strong (Samec et al. 2021; Zhai et al. 2023), the studies on asthma and ETW are contradictory. Some researchers have shown a link between ETW and asthma (Al‐Dlaigan et al. 2002; Hamasha et al. 2014; Sivasitamparam et al. 2002), while others reject this association (Gurgel et al. 2011; Rezende et al. 2019). Our study hypothesis was that the use of asthma medications correlates with the prevalence of ETW. Given the lack of data on the cause of ETW in asthmatic children, we conducted a study to aimed at determining how various asthmatic and commonly associated factors influence the prevalence of ETW in asthmatic children. We were particularly interested in the relationship between duration of asthma medication use, form of medication, the dose and frequency of medication use with ETW.

## 2 Methods

### 2.1 Ethical Aspects and Study Design

The cross‐sectional study received ethical approval from the Ethics Committee of the Ministry of Health in Slovenia (No. 165/07/09). Written informed consent was obtained from all parents for their children's participation in the study. The study population consisted of children and adolescents aged 6–17 years who were being treated for chronic bronchial asthma at the University Children's Hospital of the University Medical Centre. Inclusion criteria mandated that participants be on daily asthma medication for a minimum of 1 year and possess a medically confirmed diagnosis of asthma. Due to the lack of standardized documentation, the severity of asthma was not documented. Children with comorbid conditions such as heart disease, gastroesophageal reflux, chromosomal abnormalities, infectious diseases, eating disorders or frequent vomiting were excluded from the study, based on data collected via questionnaires completed by the parents.

### 2.2 Sample Size Calculation

According to the Statistical Office of the Republic of Slovenia, there were 229,284 children and adolescents (6–17 years old) in Slovenia in 2009. The estimated prevalence of asthma among Slovenian children is 14% (8). The calculated number of 6–17 year old asthmatic children in Slovenia was 32,100. As there is no published data on the prevalence of ETW in Slovenian asthmatic children, we used 48.6% based on a similar unpublished Slovenian study. The confidence interval was set at 95%, the margin of error at 0.05, the ETW prevalence at 48.6% and the number of asthmatic children at 32,100. A sample size of 380 participants was calculated.

### 2.3 Calibration Exercise

Calibration involved theoretical instruction, case presentations, and practical training on patients not included in the study. The intra‐ (κ = 0.95) and inter‐ (κ = 0.92) reproducibility of the investigators was assessed by examining 20 children who had not participated in the study and re‐examining them after a 2‐week interval.

### 2.4 Clinical Data Collection

Dental examinations were carried out at the university dental clinic using standard dental mirrors and ball‐ended probes under dental light by two calibrated dentists. Before examination, professional cleaning of the children's teeth was performed; however, radiographic evaluations were not included in this study. The presence of ETW was assessed using the Basic Erosive Wear Examination (BEWE) index (Bartlett et al. 2008). The buccal, occlusal, and palatal or lingual surfaces were scored: 0 = no ETW; 1 = initial loss of surface texture; 2 = clear defect, hard tissue loss < 50% of the surface; 3 = hard tissue loss ≥ 50% of the surface. All permanent teeth were evaluated except for third molars; deciduous teeth were excluded from assessment. The total BEWE score for each participant was calculated by summing the maximum values across sextants, followed by computation of the mean total BEWE score.

### 2.5 Nonclinical Data Collection

Data regarding demographics, medical history, medication usage, dietary habits, oral hygiene practices, fluoride exposure history, and specifics about asthmatic children's medication types, doses, frequencies, durations, and administration methods were collected through parent‐completed questionnaires and patient medical records. Fluoride exposure was considered using fluoride pills or use of fluoride rinse or gel. The use of acidic sports drinks was considered in the last 6 months, the exact frequency was not specified. The glucocorticoid dosage administered to children over the preceding 6 months was utilized for analysis. Frequency of medication administration was collected from patient medical records.

### 2.6 Statistical Method

The distribution of subjects among groups for categorical independent variables was analyzed using chi‐square tests. Mann–Whitney *U* tests were employed to compare differences between dependent variables and independent variables. The dependent variable (total BEWE index) was dichotomized based on its presence or absence. The total BEWE score = zero in all sextants was considered as the absence of ETW. Age was not dichotomized according to the mean value. Odds ratios (OR) and 95% confidence intervals were calculated with statistical significance set at *p* ≤ 0.05. Data analysis was conducted using SPSS version 25.0 for Windows (SPSS Inc., Chicago, IL, USA).

## 3 Results

A total of 379 asthmatic children participated in this study. The average age was recorded at 10.44 ± 2.84 years with an average duration of asthma medication usage spanning 6.01 ± 3.60 years. The prevalence rate of ETWs among participants stood at 17.2% (*n* = 379). The mean total BEWE score calculated was 0.76 ± 2.12 across a total assessment of 31,836 surfaces evaluated. Among present ETW, BEWE score 1 was the most common (72.4%), BEWE score 2 less common (25.2%) and BEWE score 3 (2.3%) rare. The distribution and characteristics of surfaces exhibiting ETW are detailed in Table 1.

**Table 1:** Characterization of surfaces with erosive tooth wear concerning presence, localization, jaw involvement, and BEWE index score.

Variable	Surfaces (*n*)	%
Surfaces with erosive tooth wear		
Present	602	1.89
Absent	31,234	98.11
Localisation of affected surfaces		
Vestibular	149	24.8
Occlusal	404	67.1
Palatine/Lingual	49	8.1
Affected surfaces in the jaw		
Maxillary	282	46.8
Mandibular	320	53.2
BEWE index score		
0	31,234	98.11
1	436	1.37
2	152	0.48
3	14	0.04
Total	31,836	100

The associations of various general and asthmatic factors with the presence or absence of ETW are presented in Tables 2 and 3. Statistically significantly higher presence of ETW was found in the group consuming acidic sports drinks, in the group aged 12–17 years, in the group using asthma medication for more than 3 years and in the group using medication in the form of dry powder inhaled form.

**Table 2:** Association between erosive tooth wear presence and different general variables (gender, fluoride exposure history, dietary history, oral hygiene, parent's education, age), *n* = 379.

Variables – general factors	Erosion present, *n* (%)	Erosion absent, *n* (%)	Total n (%)	Odds ratio (95% CI)	Statistics (Χ^2^, *p*)
**Gender**					
**Female**	25 (16.6)	126 (83.4)	151 (39.8)	0.933 (0.539–1.614)	Χ^2^ = 0.062
**Male**	40 (17.5)	188 (82.5)	228 (60.2)	1	*p* = 0.803
**Fluoride exposure history**					
**Use of fluoride pills**	25 (15.7)	134 (84.3)	159 (42.0)	0.840 (0.486–1.451)	Χ^2^ = 0.393
**No use of fluoride pills**	40 (18.2)	180 (81.8)	220 (58.0)	1	*p* = 0.531
**Use of fluoride rinse or gel**	10 (15.6)	54 (84.4)	64 (16.9)	0.875 (0.420–1.825)	Χ^2^ = 0.126
**No use of fluoride rinse or gel**	55 (17.5)	260 (82.5)	315 (83.1)	1	*p* = 0.723
**Dietary history**					
**Occasional use of chewing gum**	41 (18.6)	179 (81.4)	220 (58.0)	1.288 (0.743–2.236)	Χ^2^ = 0.815
**No use of chewing gum**	24 (15.1)	135 (84.9)	159 (42.0)	1	*p* = 0.367
**Consuming food and drinks** ≥ **5 times⁄day**	44 (18.9)	189 (81.1)	233 (61.5)	1.386 (0.786–2.443)	Χ^2^ = 1.279
**Consuming food and drinks** < **5 times⁄day**	21 (14.4)	125 (85.6)	146 (38.5)	1	*p* = 0.258
**Drinking sweet drinks between meals**	44 (17.3)	211 (82.7)	255 (67.3)	1.023 (0.578–1.810)	Χ^2^ = 0.006
**No drinking sweet drinks between meals**	21 (16.9)	103 (83.1)	124 (32.7)	1	*p* = 0.938
**Use of acid sport drinks**	**13 (37.1)**	**22 (62.9)**	**35 (9.2)**	**3.318 (1.573–6.999)**	**Χ** ^**2**^ = **10.847**
**No use of acid sport drinks**	**52 (15.1)**	**292 (84.9)**	**344 (90.8)**	**1**	***p*** = **0.001** *
**Use of sweets**	43 (20.1)	171 (79.9)	214 (56.5)	1.635 (0.934–2.861)	Χ^2^ = 2.996
**No use of sweets**	22 (13.3)	143 (86.7)	165 (43.5)	1	*p* = 0.083
**Daily use of milk and cheese**	42 (15.2)	234 (84.8)	276 (72.8)	0.624 (0.354–1.102)	Χ^2^ = 2.671
**No daily use of milk and cheese**	23 (22.3)	80 (77.7)	103 (27.2)	1	*p* = 0.102
**Oral hygiene**					
**Tooth brushing 1 time⁄day**	10 (15.2)	56 (84.8)	66 (17.4)	1	Χ^2^ = 0.225
**Tooth brushing 2 times or more⁄day**	55 (17.6)	258 (82.4)	313 (82.6)	1.194 (0.574–2.485)	*p* = 0.635
**Parents' education**					
**Elementary/profession/secondary school**	48 (17.7)	223 (82.3)	271 (71.5)	1	Χ^2^ = 0.211
**High school/university/postgraduate studies**	17 (15.7)	91 (84.3)	108 (28.5)	0.868 (0.474–1.589)	*p* = 0.646
**Age**					
**6–11 years old group**	**21 (8.2)**	**235 (91.8)**	**256 (67.5)**	**1**	**Χ** ^**2**^ = **44.442**
**12–17 years old group**	**44 (35.8)**	**79 (64.2)**	**123 (32.5)**	**6.233 (3.493–11.120)**	***p*** = **0.000** *

* *p*‐value of chi‐square test. CI, confidence interval.

**Table 3:** Association between erosive tooth wear presence and different asthmatic factors (duration of medication, mode of medication application, mouth rinse with water, use of leukotriene antagonist, antihistamines, sugar‐containing medications, spacer devices, dose, and frequency of medication), *n* = 379.

Variables – Asthmatic factors	Erosion present, *n* (%)	Erosion absent, *n* (%)	Total n (%)	Odds ratio (95% CI)	Statistics (Χ^2^, *p*)
**Duration of asthma medications use (1–3 years)**	**8 (7.3)**	**101 (92.7)**	**109 (28.8)**	**1**	**Χ** ^**2**^ = **10.368**
**Duration of asthma medications use (< 3 years)**	**57 (21.1)**	**213 (78.9)**	**270 (71.2)**	**3.379 (1.553‐7.348)**	***p*** = **0.001** *
**Medication in metered‐dose inhaled form**	**34 (13.8)**	**212 (86.2)**	**246 (69.1)**	**1**	**Χ** ^**2**^ = **10.503**
**Medication in dry powder inhaled form**	**31 (28.2)**	**79 (71.8)**	**110 (30.9)**	**2.447 (1.410‐4.245)**	***p*** = **0.001** *
**Mouth rinsed with water after medication use**	56 (18.5)	247 (81.5)	303 (79.9)	1.688 (0.794‐3.587)	Χ^2^ = 1.885
**No mouth rinsed with water after medication use**	9 (11.8)	67 (88.2)	76 (20.1)	1	*p* = 0.170
**Use of additional leukotriene antagonists**	9 (12.2)	65 (87.8)	74 (19.5)	0.616 (0.289‐1.310)	Χ^2^ = 1.610
**No additional leukotriene antagonists use**	56 (18.4)	249 (81.6)	305 (80.5)	1	*p* = 0.204
**Use of antihistamines**	2 (9.1)	20 (90.9)	22 (5.8)	0.467 (0.106‐2.048)	Χ^2^ = 1.068
**No antihistamines use**	63 (17.6)	294 (82.4)	357 (94.2)	1	*p* = 0.301
**Use of sugar‐containing medications**	33 (20.2)	130 (79.8)	163 (43.0)	1.460 (0.854‐2.494)	Χ^2^ = 1.928
**Use of nonsugar‐containing medications**	32 (14.8)	184 (85.2)	216 (57.0)	1	*p* = 0.165
**Use of inhalers with spacer**	38 (15.8)	203 (84.2)	241 (63.6)	0.770 (0.446‐1.327)	Χ^2^ = 0.891
**No spacer use**	27 (19.6)	111 (80.4)	138 (36.4)	1	*p* = 0.345
**Glucocorticoid dose (≤ 100 μg)**	29 (19.5)	120 (80.5)	149 (42.7)	1	Χ^2^ = 0.513
**Glucocorticoid dose (> 100 μg)**	33 (16.5)	167 (83.5)	200 (57.3)	0.818 (0.471‐1.419)	*p* = 0.474
**Frequency of medication administration 1/day**	30 (15.5)	164 (84.5)	194 (54.0)	1	Χ^2^ = 1.987
**Frequency of medication administration** > **1/day**	35 (21.2)	130 (78.8)	165 (46.0)	1.472 (0.858‐2.524)	*p* = 0.159

* *p*‐value of chi‐square test; CI, confidence interval.

The effects of various general and asthmatic factors and the mean total BEWE index are shown in Tables 4 and 5. Statistically significantly higher mean total BEWE indices were found in the same groups (group consuming acidic sports drinks, group of 12–17 year olds, group using asthma medication for more than 3 years, and group using medication in dry powder inhaled form).

**Table 4:** Mean total BEWE ± SD of different general variables (gender, fluoride exposure history, dietary history, oral hygiene, parent's education, age), *n* = 379.

Variables – General factors	Mean total BEWE ± SD	Total, *n* (%)	*p*‐value
**Gender**			
**Female**	0.62 ± 1.77	151 (39.8)	*p* = 0.716
**Male**	0.84 ± 2.32	228 (60.2)	
**Fluoride exposure history**			
**Use of fluoride pills**	0.74 ± 2.26	159 (42.0)	*p* = 0.555
**No use of fluoride pills**	0.76 ± 2.01	220 (58.0)	
**Use of fluoride rinse or gel**	0.44 ± 1.36	64 (16.9)	*p* = 0.575
**No use of fluoride rinse or gel**	0.81 ± 2.23	315 (83.1)	
**Dietary history**			
**Occasional use of chewing gum**	0.82 ± 2.23	220 (58.0)	*p* = 0.370
**No use of chewing gum**	0.65 ± 1.95	159 (42.0)	
**Consuming food and drinks** ≥ **5 times⁄day**	0.86 ± 2.33	233 (61.5)	*p* = 0.249
**Consuming food and drinks** < **5 times⁄day**	0.58 ± 1.70	146 (38.5)	
**Drinking sweet drinks between meals**	0.76 ± 2.12	255 (67.3)	*p* = 0.921
**No drinking sweet drinks between meals**	0.73 ± 2.10	124 (32.7)	
**Use of acid sport drinks**	**1.74** ± **3.06**	**35 (9.2)**	***p*** = **0.001** *
**No use of acid sport drinks**	**0.65** ± **1.97**	**344 (90.8)**	
**Use of sweets**	0.90 ± 2.30	214 (56.5)	*p* = 0.076
**No use of sweets**	0.56 ± 1.84	165 (43.5)	
**Daily use of milk and cheese**	0.70 ± 1.98	276 (72.8)	*p* = 0.133
**No daily use of milk and cheese**	0.91 ± 2.43	103 (27.2)	
**Oral hygiene**			
**Tooth brushing 1 time⁄day**	0.88 ± 2.32	66 (17.4)	*p* = 0.786
**Tooth brushing 2 times or more⁄day**	0.73 ± 2.07	313 (82.6)	
**Parents' education**			
**Elementary/profession/secondary school**	0.82 ± 2.23	271 (71.5)	*p* = 0.567
**High school/university/postgraduate studies**	0.58 ± 1.79	108 (28.5)	
**Age**			
**6–11 years old group**	**0.27** ± **1.20**	**256 (67.5)**	***p*** = **0.000** *
**12–17 years old group**	**1.76** ± **3.07**	**123 (32.5)**	

* *P*‐value of Mann–Whitney *U* test.

**Table 5:** Mean total BEWE ± SD of different asthmatic factors (duration of medication, mode of medication application, mouth rinse with water, use of leukotriene antagonist, antihistamines, sugar‐containing medications, spacer devices, dose and frequency of medication), *n* = 379.

Variables – Asthmatic factors	Mean BEWE ± SD	Total, *n* (%)	*p*‐value
**Duration of asthma medications use (1–3 years)**	**0.27** ± **1.12**	**109 (28.8)**	***p*** = **0.001** *
**Duration of asthma medications use (< 3 years)**	**0.95** ± **2.37**	**270 (71.2)**	
**Medication in metered‐dose inhaled form**	**0.61** ± **1.91**	**246 (69.1)**	***p*** = **0.001** *
**Medication in dry powder inhaled form**	**1.23** ± **2.62**	**110 (30.9)**	
**Mouth rinsed with water after medication use**	0.80 ± 2.18	303 (79.9)	*p* = 0.193
**No mouth rinsed with water after medication use**	0.56 ± 1.80	76 (20.1)	
**Use of additional leukotriene antagonists**	0.57 ± 1.74	74 (19.5)	*p* = 0.233
**No additional leukotriene antagonists use**	0.80 ± 2.19	305 (80.5)	
**Use of antihistamines**	0.54 ± 1.87	22 (5.8)	*p* = 0.341
**No antihistamines use**	0.76 ± 2.13	357 (94.2)	
**Use of sugar‐containing medications**	0.92 ± 2.30	163 (43.0)	*p* = 0.147
**Use of nonsugar‐containing medications**	0.62 ± 1.96	216 (57.0)	
**Use of inhalers with spacer**	0.72 ± 2.13	241 (63.6)	*p* = 0.361
**No spacer use**	0.82 ± 2.10	138 (36.4)	
**Glucocorticoid daily dose (≤ 100 μg)**	1.01 ± 2.56	149 (42.7)	*p* = 0.352
**Glucocorticoid daily dose (> 100 μg)**	0.59 ± 1.71	200 (57.3)	
**Frequency of medication administration 1/day**	0.71 ± 2.18	194 (54.0)	*p* = 0.171
**Frequency of medication administration** > **1/day**	0.89 ± 2.15	165 (46.0)	

* *p*‐value of Mann–Whitney *U* test.

## 4 Discussion

Recent literature highlights the multifaceted nature of oral health issues in patients with asthma, particularly focusing on the reduction of salivary flow attributed to beta‐agonists, the consumption of acidic beverages, the acidity of medications (especially those in dry powder form), and the implications of gastroesophageal reflux as potential contributors to ETW (Gani et al. 2020). Our findings corroborate this, revealing a statistically significant higher prevalence of ETW among children consuming acidic sports drinks, which aligns with established knowledge that acidic beverage consumption is a known risk factor for ETW (Chan et al. 2020; Pereira et al. 2020; Saads Carvalho and Lussi 2020). Notably, only 9.2% of asthmatic children in our study reported consuming such drinks, suggesting that while the risk exists, it may not be widespread.

Furthermore, our study uniquely identifies that children using asthma medication for over 3 years exhibited a higher prevalence of ETW and a greater mean total BEWE score compared to those who had been on medication for a shorter duration. This finding is consistent with previous research indicating that prolonged exposure to asthma medications may exacerbate ETW (Strużycka et al. 2017). Also, a cross‐sectional study of 400 Valencian children aged 6 to 14 years showed a statistically significant correlation with a higher BEWE index and patients using inhalers (Marqués Martínez et al. 2019).

The group utilizing dry powder inhalers also displayed a higher incidence of ETW compared to those using metered‐dose inhalers. This observation is supported by existing data indicating that dry powdered asthma medications can lower pH levels below 5.5, which is critical for hydroxyapatite dissolution (O'Sullivan and Curzon 1998). In contrast, Dugmore and Rock found no significant association between asthma and ETW in their cohort of 12‐14‐year‐old children (Dugmore and Rock 2003), highlighting the need for further investigation into this relationship.

As reported in other studies (Duangthip et al. 2018; Korkmaz and Kaptan 2020; Murakami et al. 2016), there was a statistically significant positive association between age and the presence of ETW, which was also found in our study. Although ETW is irreversible and progressive, we did not expect the presence of ETW to be this higher in the older group. We hypothesise that this difference may be due to lower parental control over adolescents and poor dietary habits. A systematic review of ETW in this group shows that carbonated drinks and consumption of acidic drinks at bedtime have the predominant erosive potential (Chan et al. 2020). Gender did not appear to influence the prevalence of ETW in our cohort; however, some studies have reported higher rates among specific demographics (Huew et al. 2012).

To mitigate the risk of ETW among children, it is imperative to implement dietary guidelines and promote the use of products that minimize demineralization while encouraging remineralization of tooth structures (Bartlett 2009). Interestingly, our study found no significant impact from fluoride exposure on the prevalence of ETW, which is partially consistent with other reviews suggesting limited efficacy of fluoride against erosive processes due to the absence of biofilm and the lower pH of erosive acids compared to bacterial acids (Huysmans et al. 2014; Lussi et al. 2019).

The mean total BEWE score in our study was relatively low compared to other similar studies (Scribante et al. 2024), which may be due to the exclusion of children with gastroesophageal reflux in our study. The prevalence rate of dental erosion among participants was 17.2%, which corresponds to a prevalence of 15% in 12‐year‐olds (Alves et al. 2015), but is relatively low compared to the prevalence of 42.3% in a slightly older population (18‐year‐olds in Poland). The distribution of BEWE scores from 1 to 3 was similar to our study (Strużycka et al. 2017).

Although the BEWE index can be used for both deciduous and permanent teeth, we have only considered permanent teeth as they have greater clinical relevance in the long term. One notable limitation of our study was the reliance on parental questionnaires for dietary intake data. Parents may not accurately report their children's consumption patterns since children also eat at school and elsewhere. Data collection through questionnaires was also limited, as no corresponding frequencies were recorded in the dietary history. While clinical examinations were utilized for detecting intraoral wear, advancements in methodologies for measuring intraoral wear are now available (Esquivel‐Upshaw et al. 2020; Longbottom et al. 2021), which could enhance future research accuracy. Examination with the BEWE index, which is considered the contemporary gold standard for ETW, is expected to be replaced by intraoral scanning in combination with a matching software that can accurately quantify clinical wear.

Children and adolescents should be discouraged from consuming acidic sports drinks, as these are associated with increased ETW. The consumption of acidic drinks should be completely replaced by the consumption of water. For asthmatic children who use dry powder for inhalation and who have been diagnosed with ETW, alternative form of medication should be discussed with their pediatrician.

## 5 Conclusion

Metered‐dose inhaled medications appear to be less detrimental to dental health compared to dry powder inhalers. Given that prolonged use of asthma medications correlates with higher rates of ETW, dentists must prioritize early detection, preventive measures, and treatment strategies aimed at mitigating ETW risks in asthmatic children.

## Author Contributions

All authors have made substantial contributions to the conception and design of the study, as well as collected and analyzed the data. Tomi Ujčič Samec led the writing.

## Ethics Statement

The study received ethical approval from the Ethics Committee of the Ministry of Health in Slovenia (No. 165/07/09). Written informed consent was obtained from all parents for their children's participation in the study.

## Conflicts of Interest

The authors declare no conflicts of interest.

## Data Availability

The data that support the findings of this study are available on request from the corresponding author. The data are not publicly available due to privacy or ethical restrictions.
